# The complete mitochondrial genome of the shoal chub, *Macrhybopsis hyostoma*

**DOI:** 10.1080/23802359.2016.1197069

**Published:** 2017-01-04

**Authors:** Sarah Gaughan, Robin Johnson, Jun Wang, Michael Wachholtz, Kirk Steffensen, Timothy King, Guoqing Lu

**Affiliations:** a Department of Biology, University of Nebraska at Omaha, Omaha, NE, USA;; b Natural Systems Analysts, Inc., Leetown Science Center, Aquatic Ecology Branch, Kearneysville, WV, USA;; c Key Laboratory of Freshwater Fisheries Germplasm Resources, Ministry of Agriculture, Shanghai Ocean University, Shanghai, China;; d Nebraska Game and Parks Commission, Lincoln, NE, USA;; e U.S. Geological Survey, Leetown Science Center, Aquatic Ecology Branch, Kearneysville, WV, USA

**Keywords:** *Macrhybopsis hyostoma*, mitochondrial, genome, next generation sequencing

## Abstract

The complete mitochondrial genome of the shoal chub (*Macrhybopsis hyostoma*) was determined to be 16,899 bp and contained 22 tRNA genes, 2 rRNA genes and 1 control region. The whole genome base composition was 30.5% A, 28.5% T, 24.9% C and 16.1 G. This complete mitochondrial genome provides essential molecular markers for resolving phylogeny and future conservation efforts.

*Macrhybopsis* chubs are a genus of the subfamily Leuciscinae, which consist of small-bodied fishes that are typically obligate river species (Galat et al. 2005). The shoal chub (*Macrhybopsis hyostoma*) serves as key food chain species for the endangered pallid sturgeon (*Scaphirhynchus albus*) (Gerrity et al. 2006; Herman et al. 2008) and has been experiencing significant population declines throughout their ranges, which may be attributable to anthropogenic disturbances (Hesse 1994; Steffensen et al. 2014). Previous molecular studies were unable to resolve the phylogeny of *Macrhybopsis* chubs with singular mitochondrial markers, making identifying populations that are susceptible to anthropogenic disturbances difficult (Nagle & Simons 2012).

Here, we report the complete mitogenome of the shoal chub, *M. hyostoma*. The shoal chub was collected from the Loup River near Pawnee Park in Columbus, Nebraska, and is part of the ichthyology collection at the University of Kansas Biodiversity Institute (KUI 41380). This mitogenome will establish a solid basis to resolve phylogenetic confusion within this genus and may aid future conservation measures.

Genomic DNA was extracted and purified from fin tissue using the Qiagen DNeasy Blood and Tissue Kit (Germantown, MD) for Genotyping by Sequencing (GBS). PCR free libraries were constructed with a TruSeq PCR Free library protocol and sequenced on an Illumina NextSeq500 (Kearneysville, WV) at the USGS Leetown Science Facility. Sequences were assembled using Velvet (Zerbino & Birney 2008), aligned with Mega 6.06 (Tamura et al. 2013) and annotated with MitoFish (Iwasaki et al. 2013). DOGMA was used to verify annotation and identify start and stop codons (Wyman et al. 2004) (Table 1).

**Table 1.** Characteristics of the mitochondrial genome of the shoal chub (*Macrhybopsis hyostoma*).

Locus	Start	Stop	Size (bp)	Start	Stop	Anticodon	Strand^a^
tRNA^Phe^	1	69	69				H
12SrRNA	70	1026	957				H
tRNA^Val^	1027	1097	71				H
16SrRNA	1098	2776	1679				H
tRNA^Leu^	2777	2852	76			UAA	H
*ND1*	2854	3828	975	ATG	TAA		H
tRNA^Ile^	3833	3904	72				H
tRNA^Gln^	3903	3973	71				L
tRNA^Met^	3975	4043	69				H
*ND2*	4044	5088	1045	ATG	TAG		H
tRNA^Trp^	5089	5159	71				H
tRNA^Ala^	5161	5229	69				L
tRNA^Asn^	5231	5303	73				L
O_L_							–
tRNA^Cys^	5334	5402	69				L
tRNA^Tyr^	5403	5473	71				L
*CO I*	5475	7025	1551	GTG	TAA		H
tRNA^Ser(UCN)^	7026	7096	71			UGA	L
tRNA^Asp^	7100	7173	74				H
*CO II*	7180	7870	691	ATG	TAA		H
tRNA^Lys^	7871	7945	75				H
*ATP8*	7947	8111	165	ATG	TAG		H
*ATP6*	8105	8787	683	ATG	TAA		H
*CO III*	8788	9571	784	ATG	TAA		H
tRNA^Gly^	9572	9643	72				H
*ND3*	9644	9992	349	ATG	TAG		H
tRNA^Arg^	9993	10,061	69				H
*ND4L*	10,062	10,358	297	ATG	TAA		H
*ND4*	10,352	11,733	1382	ATG	TAG		H
tRNA^His^	11,734	11,802	69				H
tRNA^Ser(AGY)^	11,803	11,871	69			GCU	H
tRNA^Leu(CUN)^	11,873	11,945	73			UAG	H
*ND5*	11,946	13,781	1836	ATG	TAA		H
*ND6*	13,778	14,299	522	ATG	TAA		L
tRNA^Glu^	14,300	14,371	72				L
*Cytb*	14,378	15,518	1141	ATG	TAA		H
tRNA^Thr^	15,519	15,590	72				H
tRNA^Pro^	15,590	15,659	70				L
D-loop	15,660	16,899	1240				–

^a^ H and L denote heavy and light strands, respectively.

The total length of the mitogenome was 16,899 bp (GenBank Accession No. KX139437). The mitogenomes of these two chubs consisted of 22 tRNA genes, 2 rRNA genes and 1 control region. Fourteen of the tRNA genes were encoded on the heavy (H) strand along with all of the protein-coding genes except NADH dehydrogenase subunit 6. The whole genome base composition was 30.5% A, 28.5% T, 24.9% C and 16.1 G, which is analogous to other teleost mitochondrial genomes which exhibit A/T bias (Wang et al. 2013). The putative control region was located between tRNA^Pro^ and tRNA^Phe^ and was 1,240 bp long.

To investigate the position of *M. hyostoma* within Leuciscinae, a maximum likelihood tree based on 14 complete mitochondrial genomes was constructed using MEGA6 under the GTR + G+I model with 500 bootstrap replicates (Pattengale et al. 2010; Tamura et al. 2013) (Figure 1). This maximum likelihood tree phylogenetically positioned *M. hyostoma* as a sister clade to the Notropin clade supporting previous morphological phylogenetic analysis (Cavender & Coburn 1992).

**Figure 1.**
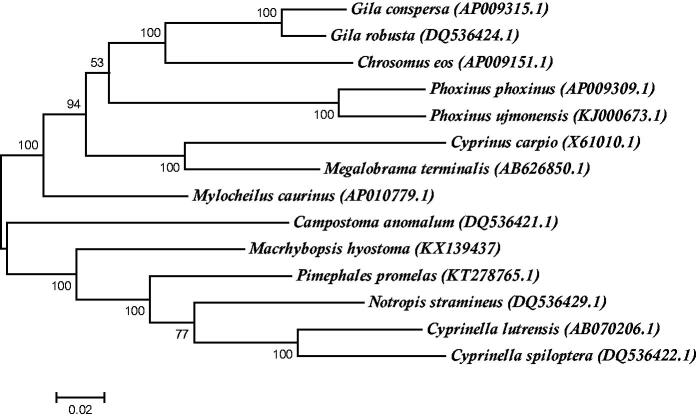
Phylogenetic tree generated using a maximum likelihood method and a general time reversal model based on fourteen complete mitochondrial genomes. The GenBank accession number is listed next to each species within the tree.
